# Tuft cells are key mediators of interkingdom interactions at mucosal barrier surfaces

**DOI:** 10.1371/journal.ppat.1010318

**Published:** 2022-03-10

**Authors:** Madison S. Strine, Craig B. Wilen

**Affiliations:** 1 Department of Laboratory Medicine, Yale School of Medicine, New Haven, Connecticut, United States of America; 2 Department of Immunobiology, Yale School of Medicine, New Haven, Connecticut, United States of America; 3 Yale Cancer Center, Yale School of Medicine, New Haven, Connecticut, United States of America; University of North Carolina at Chapel Hill School of Medicine, UNITED STATES

## Abstract

Although tuft cells were discovered over 60 years ago, their functions have long been enigmatic, especially in human health. Nonetheless, tuft cells have recently emerged as key orchestrators of the host response to diverse microbial infections in the gut and airway. While tuft cells are epithelial in origin, they exhibit functions akin to immune cells and mediate important interkingdom interactions between the host and helminths, protists, viruses, and bacteria. With broad intra- and intertissue heterogeneity, tuft cells sense and respond to microbes with exquisite specificity. Tuft cells can recognize helminth and protist infection, driving a type 2 immune response to promote parasite expulsion. Tuft cells also serve as the primary physiologic target of persistent murine norovirus (MNV) and promote immune evasion. Recently, tuft cells were also shown to be infected by rotavirus. Other viral infections, such as influenza A virus, can induce tuft cell–dependent tissue repair. In the context of coinfection, tuft cells promote neurotropic flavivirus replication by dampening antiviral adaptive immune responses. Commensal and pathogenic bacteria can regulate tuft cell abundance and function and, in turn, tuft cells are implicated in modulating bacterial infiltration and mucosal barrier integrity. However, the contribution of tuft cells to microbial sensing in humans and their resulting effector responses are poorly characterized. Herein, we aim to provide a comprehensive overview of microbial activation of tuft cells with an emphasis on tuft cell heterogeneity and differences between mouse and human tuft cell biology as it pertains to human health and disease.

## I. Introduction

Tuft cells are rare, chemosensory epithelial cells named for their characteristic tufted apical microvilli that project into the lumen of hollow organs [1]. Despite their rarity, tuft cells have been found in the respiratory tract, gastrointestinal tract, urogenital tract, and thymus at varying levels of abundance [2–13]. In the respiratory tract, tuft cells compose 1% to 10% of the upper airway mucosal epithelium but are absent in the lower airway until tissue damage [3,14]. In the intestinal tract, tuft cells constitute approximately 0.4% to 2% of the epithelium [15,16]. Tuft cell numbers are elevated in biliary and pancreatic tracts, comprising 20% to 30% of the epithelium in these sites [10,17]. Based on their morphology in these locations, tuft cells have amassed many names, including brush and solitary chemosensory cells (airway), caveolated cells (stomach), and multi- or fibrillovesicular cells (olfactory epithelium) [3,18–20]. The evolutionary origin of tuft cells is unknown, but they are widespread across vertebrate species, including placental mammals, snakes, and bullfrogs [3,11,21]. Developmentally, tuft cells require the transcription factor POU Class 2 Homeobox 3 (POU2F3) for differentiation in diverse mucosal surfaces and the thymus [15,22–25]. In the gastrointestinal tract where tuft cells are best characterized, tuft cells are terminally differentiated cells that arise from Leucine Rich Repeat Containing G Protein–Coupled Receptor 5 (Lgr5+) intestinal stem cells in the crypt [15,26]. While the exact environmental cues and transcriptional regulation that drive this process are not completely clear, it has been shown that immune cell–derived cytokines such as interleukin (IL)-13 act on Lgr5+ stem cells to drive polarization toward the tuft cell lineage [27–30]. Other proteins, such as the Taste 1 Receptor Member 3 (TAS1R3) and mechanistic target of rapamycin (mTORC1), also regulate homeostatic tuft cell differentiation and abundance [31,32]. Whether tuft cells emerge from a secretory (Atonal BHLH Transcription Factor 1 (ATOH1-dependent) or nonsecretory (ATOH1-independent) lineage progenitor may vary by anatomic location [15,33–35].

**Fig 1:**
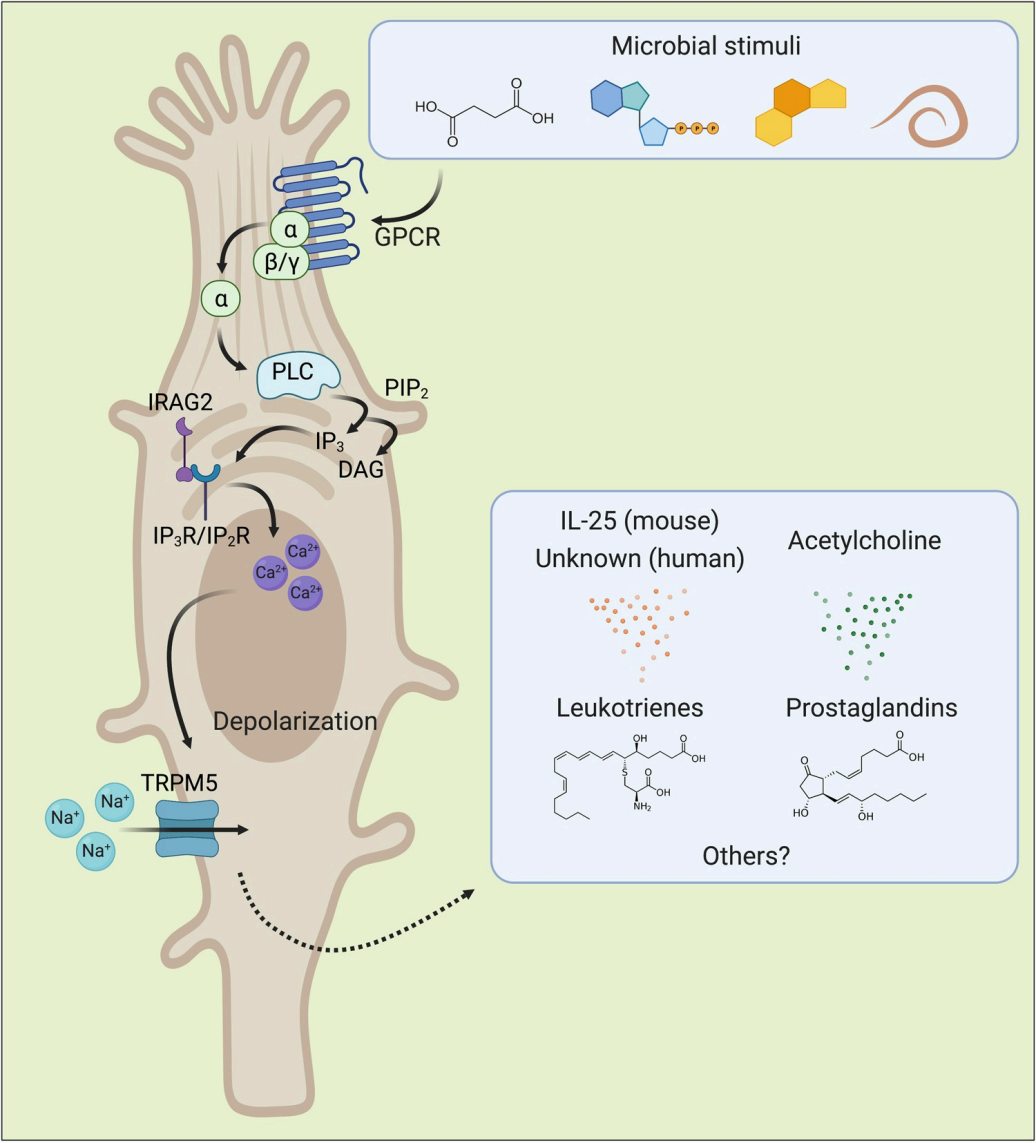
Canonical tuft cell ligands, downstream signal transduction, and effector molecules as understood to date in mouse and human tuft cells. Molecules derived or produced by microbes and damage-associated by-products of microbial infection can drive tuft cell activation through GPCRs. Most commonly, the GPCR alpha subunit GNAT3 activates PLCβ2 and promotes this signaling cascade after receptor binding, but other alpha subunits and PLC family members have also been implicated. It is not known whether all responses share downstream intracellular signaling pathways, but evidence suggests there may be pathogen-specific and location-specific differences. The transcriptional regulation driving expression of tuft cell signaling components is poorly characterized, but it is known that p53 regulates expression of the transmembrane protein coding gene *Irag2*, which is required for calcium flux. Additional molecules that activate tuft cells likely exist that have not been discovered [23,29,31,40,43,47,49–55]. GNAT3, G Protein Subunit Alpha Transducin 3; GPCR, G protein–coupled receptor; IRAG2, Inositol 1,4,5-Triphosphate Receptor Associated 2; PLCβ2, phospholipase C beta 2.

Since their original discovery in the rat trachea and mouse stomach in 1956, the functional roles of tuft cells have remained elusive for over 60 years, even after their identification in humans [2,4,36]. The general signal transduction pathways and effector biosynthetic pathways within tuft cells have been extensively reviewed elsewhere and are outlined generally in Fig 1 [1,37–39]. Despite different sensor and effector functions, tuft cells share many signal transduction pathways with type II taste cells [37]. Briefly, ligands bind to G protein–coupled receptors (GPCRs) on the surface of tuft cells. Intracellular G protein alpha subunits are subsequently activated and promote cleavage of the membranous lipid phosphatidylinositol 4,5-bisphosphate (PIP_2_) by phospholipase C beta 2 (PLCβ2) or another PLC family member, producing diacylglycerol (DAG) and inositol triphosphate (IP_3_). IP_3_ then binds its cognate receptor IP_2_R in the small intestine or IP_3_R in the airway and causes calcium efflux from the endoplasmic reticulum. Calcium efflux also appears to require interactions between Inositol 1,4,5-Triphosphate Receptor Associated 2 (IRAG2) with IP_3_ receptors [40]. Intracellular calcium flux activates transient receptor potential cation channel subfamily M member 5 (TRPM5), which allows sodium influx and subsequent cellular depolarization. This calcium flux and depolarization are canonical measures of tuft cell activation. Downstream of TRPM5, undefined pathways trigger the release of the tuft cell effectors IL-25, acetylcholine (ACh), leukotrienes (LTs), and prostaglandins (PGs) (Fig 1) [29,41–46]. The secretory systems that control effector release are not well understood, and their regulation requires further research. It is known that release of IL-25 can be blocked with the vesicular transport inhibitor Brefeldin A, suggesting that it may be stored in vesicles until triggered for vesicular release [47]. ACh, in contrast, may be secreted in a noncanonical mechanism, as tuft cells do not detectably express the machinery required for ACh to undergo synaptic release [48].

Based on their known signaling pathways, tuft cells have been linked to a wide variety of bodily functions, such as the establishment of T cell tolerance, cross talk with the nervous system, epithelial repair and remodeling, cell division, and luminal sensing of microbes [9,24,56–60]. Given these roles, it is unsurprising that tuft cells have been implicated in allergy, inflammatory bowel disease (IBD), and cancer [25,44,45,60–71]. Tuft cells are suggested to have protective functions in the 2 manifestations of IBD: Crohn disease and ulcerative colitis [60,62–64]. The specific mechanisms by which tuft cells mitigate IBD have not been elucidated, but this may be microbiome dependent. Perhaps tuft cells regulate microbiota populations and support protective mucosal barrier function, ameliorating the dysbiosis and bowel dysfunction observed in mouse models and IBD patients. As in IBD, tuft cells may possess protective roles against cancer, such as by impeding Kras-mediated pancreatic tumorigenesis via the production of prostaglandin D_2_ (PGD_2_) [45]. In a seemingly juxtaposed role, a number of cancers (colon, pancreatic, thymic, gastric, and small cell lung cancer) have been evidenced to arise from tuft cell origin or to be mediated at least in part by tuft cells [25,65–73]. Further investigation on these topics is warranted, as our understanding of the specific mechanisms by which tuft cells modulate cellular behavior to prevent or promote disease is incomplete.

The best described function of tuft cells emerged after their recent implication in immune sensing of helminths and protists. Through use of the canonical signal transduction pathways delineated in Fig 1, tuft cells can sense the luminal environment. In responding to these microbes at steady state and during insult, tuft cells boast diverse functions ranging from regulating immunity, driving epithelial repair, and maintaining homeostasis. In the mouse small intestine, parasites can trigger tuft cells to release IL-25, stimulating type 2 innate lymphoid cells (ILC2s) to produce IL-5/9/13. These cytokines then drive a type 2 immune response—an inflammatory adaptive immune response that is classically associated with allergy and parasitic infection—which then skews epithelial differentiation [23,29,43,47,49–52,64]. Interestingly, the upstream signaling molecules that drive this pathway differ depending on the pathogen, suggesting unique specificity to tuft cell–mediated immune responses. Tuft cells in the mouse upper airway and lung similarly mediate this type 2 immune pathway, reacting to aeroallergens and transitory helminth infection, respectively [44,55,74,75]. Tuft cell involvement in type 2 immunity has also been reported in the context of coinfection, where tuft cell–IL-4 circuits can impair virus-specific CD8+ T cells during concurrent helminth and viral infection [76,77]. Unlike the small intestine, tuft cells in the colon do not perform parasite-driven tuft cell–ILC2 circuits and instead respond to bacteria. Bacterial microflora can regulate colonic tuft cell numbers and induce tuft cell expansion, and colonic tuft cells have been reported to reduce bacterial infiltration and facilitate epithelial repair [16,60,64,78]. In both the small and large intestine, tuft cells serve as the primary physiologic target of persistent murine norovirus (MNV) viral infection and may promote immune evasion [78–80]. Ultimately, tuft cells respond to both commensal and pathogenic microorganisms, and they perform heterogeneous immunomodulatory functions that vary by anatomic location. This review aims to provide a comprehensive overview of microbial activation of tuft cells with an emphasis on tuft cell heterogeneity and differences between mouse and human tuft cell biology as they relate to human health and disease.

## II. Tuft cell heterogeneity

Tuft cells express several characteristic markers that distinguish them from other cell types (Table 1). Tuft cells exhibit broad inter- and intracellular diversity with variation in their expression profiles, phenotypic behavior, and development. While tuft cell receptor and effector gene expression may differ by anatomic location, only a small fraction of these transcriptional differences have been functionally mapped. For example, in the thymus, in addition to their canonical markers, tuft cells also uniquely express antigen presentation hallmarks such as *L1cam* and genes that encode for major histocompatibility complex II proteins (*H2-Aa*, *H2-Ab*, and *CD74*) that support their function in thymocyte development and immune tolerance [9,52]. Despite their conservation among mammals, tuft cells also exhibit distinct differences in both marker profiles and phenotypic behavior between mice and humans (Table 1).

**Table 1:** Expression of canonical tuft cell markers across anatomical locations and tuft cell subsets in *Mus musculus* and *Homo sapiens*.

Marker	Name	*M*. *musculus*	*H*. *sapiens*	Expression patterns and exceptions	Source(s)
DCLK1	Doublecortin-like Kinase 1	*✓*		Most tuft cells (>95%)	[1,64,81]
POU2F3	POU Class 2 Homeobox 3	*✓*	*✓*	All tuft cells	[23–25]
GFI1B	Growth Factor Independent 1B Transcriptional Repressor	*✓*		All tuft cells	[22,34]
AVIL	Advillin	*✓*	*✓*	Intestinal tuft cells	[82,83]
ALOX5AP	Arachidonate 5-Lipoxygenase Activating Protein	*✓*	*✓*	All tuft cells	[82,83]
ALOX5	Arachidonate 5-Lipoxygenase	*✓*	*✓*	All tuft cells	[43,82]
PTGS1 (COX-1)	Prostaglandin-Endoperoxide Synthase 1 (Cyclo-oxygenase-1)	*✓*	*✓*	All tuft cells	[82,83]
PTGS2 (COX-2)	Prostaglandin-Endoperoxide Synthase 2 (Cyclo-oxygenase-2)	*✓*	*✓*	All tuft cells	[16,82]
HPGDS	Hematopoietic Prostaglandin D Synthase	*✓*	*✓*	Small intestinal tuft cells	[15,83]
IL-25	Interleukin-25	*✓*	*	All tuft cells*	[22,29,84–86]
PLC*β*2	Phospholipase C Beta 2	*✓*		Most tuft cells; skewed toward tuft-1 populations	[87,88]
ChAT	Choline O-Acetyltransferase	*✓*	*✓*	Most tuft cells; not in type II taste bud cells	[46,83,89,90]
SIGLECF	Sialic acid-binding Immunoglobulin-like Lectin F	*✓*		Intestinal and pancreatic tuft cells	[22,23]
pEGFR	Epidermal Growth Factor Receptor	*✓*	*✓*	All tuft cells	[16]
GNAT3	G Protein Subunit Alpha Transducin 3	*✓*		Most tuft cells, skewed toward tuft-1 populations	[22,91]
TAS2Rs and TAS1Rs	Taste 2 Receptors and Taste 1 Receptors	*✓*	*✓*	Lowly/undetectably expressed in the intestinal tract; specific receptor expression and combinations may vary by tissue; skewed toward tuft-1 populations	[31,47,52,88]
TRPM5	Transient Receptor Potential Cation Channel Subfamily M Member 5	*✓*	*✓*	All tuft cells	[82,92]

*IL-25 transcripts have been detected in diseased nasal epithelium of humans but have not been identified in tuft cells in other contexts [22,84–86].

### Transcriptomic analyses reveal distinct tuft cell subsets

Single-cell RNA sequencing (scRNA-seq) analyses have uncovered distinct tuft cell subsets. Tuft cells cluster separately by expression profile both within and between anatomic locations [52]. Some studies have found 2 distinct mature tuft cell populations within a given site deemed “tuft-1” and “tuft-2” [93,94]. In the small intestine, tuft-1 and tuft-2 populations share consensus tuft cell signatures and express *Dclk1*, *CD24*, *Pou2f3*, *Trpm5*, *a*nd *IL25* [93]. In the airway, canonical consensus tuft cell markers are also shared but some skew more toward tuft-1 (*Pou2f3* and *Gnat3*) or tuft-2 (*Gfi1b*, *Alox5ap*, *Sox9*, *Dclk1*, and *CD24*) populations [52,94]. In general, tuft-1 populations express tuft cell–specific genes related mostly to taste transduction, such as *Tas2r* and *Tas1r* gene families, *Gnat3*, and *Plcb2*, although taste receptor expression is limited in intestinal tuft cells [52,93,94]. Tuft-2 populations overall surprisingly express immune-related genes, including *Ly6e*, *TSLP*, and even the hematopoietic lineage marker *Ptprc* (CD45) [93,94]. However, not all single-cell sequencing analyses have unveiled tuft-1 and tuft-2 populations and instead identify tuft cells as a single cluster [22,35,52,64]. Within their cluster, some of these tuft cells are still heterogeneous in their expression of specific effector pathway components. For instance, only a subset of tuft cells expresses detectable *Fcgr2a* (the Fc receptor activated by IgG) or the vesicular ACh transporter required for ACh trafficking and secretion [22,48]. In response to cholinergic blockade, some tuft cells can expand and adopt an enteroendocrine transcriptional profile, releasing more ACh to restore cholinergic homeostasis [95].

### Long- and short-lived tuft cells

Various studies have demonstrated that mature tuft cells transit upward along the crypt-villus axis as terminally differentiated cells [15,96,97]. With a half-life of approximately 3 to 4 days, tuft cells are generally described as short-lived, postmitotic cells that continually undergo turnover in the intestinal epithelium. A small subset of tuft cells may exhibit long-lived, quiescent stem cell-like behavior with self-renewal properties. This long-lived subpopulation may constitute 5% of the overall tuft cell population and is characterized by the expression of pluripotency factors (*Oct4*, *Nanog*, *Klf4*, and *Sox2*), survival factors (Survivin, *Akt*, and p53), and markedly low turnover rates [66,98]. Although DCLK1 is expressed at the +4 position of the crypt, a known site of quiescent intestinal stem cells, long-lived tuft cells express other tuft cell–specific markers, like COX2, and likely do not function as reserve stem cells [66,98–102]. Instead, their expression profiles poise them for injury repair and uncontrolled proliferation. Upon inflammatory insult, tuft cells can serve as tumor stem cells in colorectal cancer and some colorectal tumors can originate from long-lived tuft cells [66,97]. Whether long-lived tuft cells are present in all mice and in extraintestinal sites is unknown. Further, the presence of long-lived tuft cells in humans has yet to be confirmed, although their existence may have important ramifications in human cancers. In mice, long-lived tuft cells have been traced using Cre recombinases driven by expression of the mouse-specific tuft cell marker *Dclk1* [35,66,98,103]. Therefore, probing for a comparable tuft cell subset in humans will likely require an alternative cognate tuft cell marker.

### Murine versus human tuft cells

Murine and human tuft cells share similar structure and anatomical distribution, although tuft-1 versus tuft-2 populations and the presence of tuft cells in the cecum have yet to be described in humans [1,56,83]. Some human tuft cell functions that mirror those of mice have also been identified, including their role in thymocyte development and potentiating type 2 immune circuits [75,85,104]. However, IL-25 production of human tuft cells has only been described in the nasal epithelium [84–86]. IL-25 transcripts are not robustly detected in tuft cells from intestinal tissue in nonhuman primates or humans [22,42]. Whether human tuft cells produce an alternative effector molecule instead of IL-25 and under what circumstances human tuft cells promote type 2 immunity is an active area of investigation. In addition to IL-25, human and mouse tuft cells exhibit differential expression of some canonical tuft cell markers, most notably DCLK1, but how these differences affect tuft cell functionality is unknown (Table 1) [64,103]. With a heavy reliance on mouse models, our understanding of human tuft cell biology remains impeded by the rarity of tuft cells in human scRNA-seq analyses, limited genetic modeling tools, and the fact that many human samples are derived from diseased patients. Nonetheless, translating these key findings to humans is an important future direction.

## III. Tuft cells in host–microbe interactions

Located in the mucosal epithelium, tuft cells are subject to continual microbial exposure and insult. It is thus unsurprising that tuft cells have evolved to perform critical functions in modulating host–microbe interactions. Tuft cells are best characterized as type 2 immune mediators, specifically during parasitic infection, and the role of tuft cells in other microbial interactions remains an active area of investigation. Tuft cells function as luminal sentinels that can sense and respond to a variety of microbial stimuli beyond parasitic helminths and protozoa, including bacteria, viruses, and fungi. In each anatomic location, tuft cells have adaptively tuned their responses to each class of microbe with exquisite specificity, potentially in part due to their heterogeneity. Still, it is poorly understood how many of these interactions are regulated and how specific tuft cell subpopulations differentially contribute to microbial sensing. Importantly, it is even less understood how many these interactions translate to human health, as the majority of tuft cell functions associated with luminal perturbations have been studied only in small animal models.

### Parasites (protists, protozoa, and helminths)

It was long appreciated that IL-25 is one of the earliest cytokines induced in response to helminth infection, but the cells that sense helminths and produce IL-25 were unknown until the simultaneous discovery that tuft cells were the elusive source [23,29,49]. Infection with the transitory helminth *Nippostrongylus brasiliensis* induces a strong type 2 immune response driven by IL-25 and IL-13 that facilitates worm clearance within 7 to 10 days [23,49]. By employing an IL-25 reporter mouse known as Flare25 (flox and reporter of *IL25; IL25*^*F25/F25*^), tuft cells were found to constitutively express IL-25, implicating them as possible mediators of type 2 immunity [29]. At resting state, tuft cell–derived IL-25 acts in a paracrine manner on the IL-25 receptor IL17RB on ILC2s to support homeostatic production of IL-13 [29]. IL-13 in turn acts on IL4RA/IL13RA dimers on epithelial progenitors in the crypt, regulating intestinal homeostasis and supporting tuft cell and goblet cell differentiation [23,29]. During *N*. *brasiliensis* infection, this feed-forward loop becomes more pronounced, inducing a 10- to 15-fold increase in tuft cell abundance and small intestine lengthening [23,29,49–51]. Tuft cell activation by *N*. *brasiliensis* requires expression of TRPM5, ILC2 production of IL-13, downstream signaling through IL4RA, and Signal Transducer and Activation of Transcription 6 (STAT6) [23,29,49,50,52]. Despite its role in signaling, GNAT3 is not required for tuft cell sensing of *N*. *brasiliensis* [52]. While recombinant IL-4 and IL-13 can drive this circuit, endogenous IL-4 is unaffected by loss of tuft cells and is dispensable for in vivo *N*. *brasiliensis* infection [23]. This same positive feedback loop has been identified during infection with the protozoan *Tritrichomonas muris* and the intestinal helminths *Trichinella spiralis*, *Heligmosomoides polygyrus*, and *Hymenolepis microstoma*, although the degree of tuft cell hyperplasia may vary [29,49,53,93]. In addition to IL-25, helminths trigger small intestinal tuft cells to secrete cysteinyl leukotrienes (CysLTs) [43]. The CysLTs LTC_4_ and LTD_4_ bind CysLTR1 on the surface of ILC2s, likely activating ILC2s via induction of Nuclear Factor of Activated T-cell (NFAT) signaling [43,74]. CysLTs constitute a critical and nonredundant component of the anti-helminth immune response, as they operate synergistically with IL-25 signaling and worm clearance is significantly delayed in their absence [43]. Surprisingly, the type 2 immune response against intestinal protists does not require CysLTs, indicating that tuft cell effectors can differ between microbial taxonomies within the same tissue [43]. Much like CysLTs, ACh is indispensable for driving optimal tuft cell–ILC2 immune responses [105]. *N*. *brasiliensis* and *T*. *muris* can induce ChAT expression and ACh production by ILC2s, and IL-25 up-regulates ACh receptors on ILC2s [105]. In the absence of ILC2-derived ACh acting in an autocrine loop, helminth expulsion is significantly dampened [105]. Whether tuft cell–derived ACh acts directly on ILC2s is not clear. Tuft cells also up-regulate *Hpgds2*, *Cox1*, and *Cox2* and produce PGD_2_ during late *N*. *brasiliensis* infection [106]. Tuft cell–derived PGD_2_, unlike CysLTs and ACh, acts as a negative regulator of tuft cell–ILC2 immunity by down-regulating *Il13ra* and limiting intestinal stem cell differentiation programs driven by type 2 immune cytokines [106]. Ultimately, PGD_2_ seems to aid in restoring intestinal cell populations to homeostatic baseline after infection [106]. Whether this mechanism is true for all intestinal parasites is not known and requires further investigation.

The tuft cell–ILC2 type 2 circuit is present but restrained in the absence of infection, requiring an activating trigger from parasitic infection. For *T*. *muris and* other related Tritrichomonad species, the activating signal has been identified as the fermentative end product and tricarboxylic acid (TCA) cycle intermediate succinate [51,52]. Succinate activates tuft cells directly by binding to the GPCR succinate receptor 1 (SUCNR1/GPR91) and initiating a GNAT3-dependent signal cascade that drives IL-25 release in a TRPM5-dependent manner [51,52]. The tuft cell receptor TAS1R3 also facilitates a type 2 immune response against *T*. *muris* colonization. Although TAS1R3 is not required for tuft cells to sense succinate, genetic ablation of *Tas1r3* dampens tuft cell hyperplasia. Increased duration of succinate treatment can overcome a TAS1R3 deficiency, suggesting that while TAS1R3 is not required for tuft cells to sense succinate, this receptor may potentiate the response or improve tuft cell response kinetics [31].

Unlike *T*. *muris*, tuft cell activation by the helminths *N*. *brasiliensis*, *T*. *spiralis*, and *H*. *polygyrus* is SUCNR1- and TAS1R3 independent [47,50,52]. These helminths preferentially colonize the proximal small intestine, where *Sucnr1* expression is relatively low. In contrast, *T*. *muris* colonizes the SUCNR1-rich distal small intestine [43,51,52]. Tuft cells in the proximal small intestine are less responsive to succinate as a result, and tuft cells have adapted alternative strategies to mount immune responses against these parasites [43,51,52]. In the case of *T*. *spiralis*, excretory–secretory (E–S) products and worm extracts activate tuft cells by acting on the bitter taste receptor TAS2R143 [47]. The specific molecule(s) from *T*. *spiralis* that trigger tuft cell activation and whether additional TAS2R family members are involved in initial *T*. *spiralis* sensing are undetermined [47]. To date, the identity and nature of the activating signal(s) in *N*. *brasiliensis* and *H*. *polygyrus* colonization remain unknown. Whether *N*. *brasiliensis* activates this circuit by first directly activating tuft cells or indirectly by triggering ILC2s is not clear. In either case, it is likely that *N*. *brasiliensis* activates tuft cells by introducing an activating ligand or removing an inhibitory signal that propels this circuit forward. Direct positive and negative regulators of tuft cell function remain obscured, but multiple negative regulators of ILC2-mediated type 2 immune responses have been described. For example, the E3 ubiquitin ligase A20 acts as a negative regulator of IL17RB on ILC2s and that A20 deficiency can spontaneously trigger this type 2 immune loop [51]. A20 is notably down-regulated during *N*. *brasiliensis* infection [51]. Cytokine-inducible SH2-containing protein (CISH) also negatively regulates ILC2s at homeostasis and during *N*. *brasiliensis* infection, as CISH restricts IL-25–dependent ILC2 activation [107]. Global or ILC2-specific CISH knockout also expedites *N*. *brasiliensis* expulsion and increases tuft cell numbers at early infection time points [107]. Perhaps *N*. *brasiliensis* interferes with A20, CISH, or other negative regulators of ILC2s to release the brakes on this type 2 immune loop.

Whatever the activating signal, tuft cell activation is characterized by a series of intracellular signaling cascades that proceed as outlined in Fig 1. This pathway has been best defined during *T*. *spiralis* infection, where TAS2R activation drives tuft cell trimeric G proteins Gα-Gustducin/Gβ1γ13 and/or Gαo/Gβ1γ13 to dissociate and activate PLCβ2 to cleave IP_3_, which drives calcium release from the endoplasmic reticulum via binding at IP_3_R2 [47]. Calcium release then induces TRPM5 to open, causing cellular depolarization that stimulates IL-25 release—pushing the classical tuft cell–ILC2 loop into action [47]. Downstream mucosal type 2 immune responses aid in mounting a classical “weep and sweep” response and gasdermin C–mediated activation that drive helminth expulsion [23,108].

Parasitic infections by *N*. *brasiliensis*, *T*. *spiralis*, and *H*. *polygyrus* are typically self-limiting with rapid clearance mediated by the tuft cell–ILC2 circuit. However, unlike *N*. *brasiliensis* and *T*. *spiralis*, tuft cell hyperplasia is markedly lower (approximately 5- to 10-fold) during *H*. *polygyrus* infection and downstream immune-mediated clearance takes months [23,109]. Recently, it was shown that *H*. *polygyrus* E–S products suppress tuft cell differentiation, and this likely has critical consequences for tuft cell–mediated control of chronic helminth infection [109]. With the prevalence of endemic helminth infection in human populations, the potential role of human tuft cells in intestinal type 2 immunity and chronic/long-term or secondary helminth infection are of vital interest. How the tuft cell–ILC2 circuit translates to repeated helminth infection or endemic helminth infection in the small intestine is currently unexplored.

### Bacteria

During pathogenic bacterial infection with *Salmonella enterica*, tuft cells in the small intestine do not undergo significant transcriptional changes, suggesting that they may not respond to bacteria [93]. A similar effect has been observed with commensal microbes in the small intestine, where tuft cells are generally resistant to transcriptional perturbation by antibiotic-mediated depletion of the gut microbiome [64,78]. However, evidence thus far suggests that tuft cells in the small intestine can sense some bacteria such as members of the *Bifidobacterium* genus in a succinate-dependent manner [64]. In response to bacterial succinate, ATOH1-independent ileal tuft cells initiate the same type 2 immune circuit driven by Tritrichomonads via SUCNR1 and up-regulate a variety of TCA genes to modulate inflammation in the small intestine [50,64]. The precise mechanisms by which succinate alters tuft cell gene expression and the consequences of small intestinal tuft cell hyperplasia on the microbiome are unclear.

In contrast to the small intestine, ATOH1-dependent colonic tuft cells are highly sensitive to intestinal bacteria and respond independently of SUCNR1 or type 2 immune circuits. After exposure to antibiotics, tuft cells in the large intestine are significantly depleted [78]. Moreover, the microbiome can causes changes in colonic tuft cell gene expression and tuft cell expansion, as evidenced by helminth-free fecal gavage in germ-free mice [16]. Tuft cell expansion under these conditions is mitigated after 8 weeks, and tuft cell numbers then return to baseline [16]. Furthermore, when the mucosal barrier is breached by bacteria, tuft cell differentiation is stifled, and tuft cell numbers decrease [110]. Previous work has shown that tuft cells are critical for promoting epithelial repair and limiting this bacterial infiltration, especially in bacterial-induced colitis [60]. Together, these findings imply balanced bidirectional regulation, whereby the intestinal microbiome maintains homeostatic colonic tuft cell populations and tuft cells prevent bacterial infiltration and dysbiosis via unidentified direct or indirect mechanism(s). As tuft cells have been linked to IBD in humans, the interactions of tuft cells with the bacterial microbiome may have implications for human health [62].

A similar phenomenon has been described in the airway of mice and humans, where formylated peptides, gram-negative quorum sensing molecules (QSMs, e.g., acyl homoserine lactones), and some D-amino acids can activate tuft cells [41,57,89,111–113]. While the receptors for formylated peptides and QSMs have not been identified, D-amino acids appear to trigger tuft cells using the sweet taste receptor TAS1R2/3 [41,57,89,111–113]. The functional consequence of these interactions is poorly characterized, but they may alter immune responses against pathogenic bacteria. It was previously shown that calcium flux originating from upper airway tuft cells can propagate through gap junctions and trigger antimicrobial peptide (AMP) secretion from neighboring epithelial cells [114]. Importantly, *Staphylococcus*-derived D-amino acids can impair antibacterial innate immune responses by reducing AMP production or release [113]. In contrast, QSMs can incite tuft cells to produce ACh, which may facilitate mucociliary clearance of virulent bacteria [41,57].

### Viruses

Tuft cells can mediate virus pathogenesis both directly by serving as a target cell for infection and indirectly by coinfection driven immune-mediated effects. Both MNV and murine rotavirus can directly infect and replicate within tuft cells, whereas helminth promotion of West Nile virus (WNV) pathogenesis requires the immunomodulatory functions of tuft cells. Tuft cells express the MNV receptor CD300lf and are the primary physiologic target for the persistent strain MNV^CR6^ [78]. Inducing tuft cell hyperplasia in the small intestine with type 2 cytokines promotes MNV^CR6^ infection, while reducing colonic tuft cells with broad-spectrum antibiotics is associated with resistance to MNV^CR6^ infection [78,79]. Tuft cell tropism also enables MNV^CR6^ evasion of the adaptive immune system. While CD8+ T cells activated by MNV^CR6^ appear functional, they fail to eliminate infected tuft cells. Similarly, MNV^CR6^ elicits a neutralizing antibody response, but this is not sufficient to clear infection from tuft cells [115]. This suggests tuft cells may serve as an immune-privileged niche that promotes norovirus immune escape [78,80]. Furthermore, MNV^CR6^ infection of tuft cells in germ-free mice restores disrupted barrier integrity, regulates immune cell populations, and supports intestinal homeostasis [116]. Taken together, these findings demonstrate that tuft cells are critical for intestinal epithelial homeostasis and immunity. Whether tuft cells mediate human norovirus infection and pathogenesis remains unclear. The receptor for human norovirus remains unknown, but human CD300lf does not appear to act as a receptor [117]. Intestinal epithelial cells including enteroendocrine cells support human norovirus infection and B cells support replication of the human norovirus strain GII.4-Sydney (genogroup II, genotype 4, Sydney isolate) [118–120].

Very recently, it was demonstrated that murine rotavirus also infects tuft cells [121]. While rotavirus primarily infects mature enterocytes near the apical villi of the small intestine, infected tuft cells were identified by scRNA-seq and immunofluorescence [121]. More research will be required to clarify whether rotavirus productively infects tuft cells or whether tuft cells employ any postentry barriers that restrict rotavirus replication. In the rotavirus-infected epithelium, tuft cells up-regulate interferon stimulated genes and a number of viral defense pathways, suggesting they can effectively sense enteric viral infection [121]. During rotavirus infection, tuft cell responses are categorically different than those seen during parasitic infection. In the rotavirus-infected epithelium, tuft cells down-regulate *Il25*, *Alox5*, *Alox5ap*, and *Ltc4s* and up-regulate *Trpm5*, *Plcb2*, and *Plcg2* [121]. The functional consequence of up-regulating intermediate signaling genes while simultaneously down-regulating downstream effector genes is unclear, but this finding suggests that tuft cells may perform divergent chemosensory pathways in the presence of enteric viral infection. Maybe other chemosensory effectors are secreted in response to rotavirus, as TRPM5 and PLC family proteins are essential for tuft cell activation. How tuft cells contribute to rotavirus pathogenesis and how tuft cells offer an immune privileged niche for some viruses but not others remain open questions.

In the context of coinfection, tuft cells also potentiate viral infection. During coinfection with the helminth *T*. *spiralis* and MNV^CR6^, tuft cells populations expand and virus-specific CD8+ T cell populations trend modestly downward, which is associated with increases in MNV^CR6^ viral load during persistent infection [76,78]. A similar phenotype has been described during coinfection with helminth *H*. *polygyrus bakeri* during infection with the flaviviruses WNV, Zika virus, and Powassan virus [77]. During flavivirus and helminth coinfection, this tuft cell–IL-4 circuit acts directly on the intestinal epithelium to impair virus-specific CD8+ T cell survival, enabling higher viral replication in multiple segments of the gastrointestinal tract and central nervous system [77]. Taken together, these findings suggest that tuft cells may modulate viral persistence and the CD8+ adaptive immune response. Despite high rates of norovirus and flavivirus infection in countries with endemic helminth burden and previous reports of coinfection, the role of tuft cells in helminth–virus coinfection in humans is unclear [122–124].

Viral infection can also prompt de novo tuft cells development. At steady state, tuft cells are not present in the alveolar epithelium of the lower airway [14]. After infection with the influenza A subtype H1N1, the lung undergoes dramatic dysplastic remodeling and tuft cells appear de novo at approximately 25 to 51 days postinfection [14]. The functional role for these tuft cells is unknown, but it has been suggested that they may facilitate injured lung tissue to rapidly respond to damage signals [14]. Much like in tuft cell type 2 immune feed-forward circuits, tuft cells that appear after lung damage appear promote and reinforce their own expansion [14]. The proposed the role of tuft cells in lung inflammation and repair is poorly explored across other respiratory viruses, although a modest 3-fold increase in tuft-like cells in the upper airway and ectopic development of tuft-like cells in the lung has been recently described in human patients with Coronavirus Disease 2019 (COVID-19) [125]. The physiological relevance and activities of these tuft cells merit further investigation in virus-induced lung injury, but they may contribute to pathophysiology or tissue repair.

### Other

Tuft cells in the upper airway have been implicated in type 2 immune responses, especially after stimulation with aeroallergens. Exposure to fungal chitin, dust mites, or *Alternaria* mold can elicit the release of ATP, a typical damage-associated molecular pattern (DAMP). ATP release triggers the purinergic P2Y2 receptor on tuft cells and stimulates tuft cells to release IL-25 and CysLTs like LTE_4_ [44]. LTE_4_ subsequently binds the high affinity receptor CysLT_3_R on tuft cells while other CysLTs bind their cognate receptors on ILC2s, driving a feed-forward type 2 immune circuit similar to that of small intestine [44,55]. Although other LTs can activate ILC2s directly, LTE_4_ may indirectly activate ILC2s via tuft cell release of IL-25 in vivo, as LTE_4_ poorly binds other CysLTRs and ILC2s lack CysLT_3_R expression [55,74]. While this pathway is IL-25–dependent in the upper airway, it likely operates in a STAT6-independent manner, unlike in the intestinal tract [55]. In humans, *Aspergillus* fungus and *Alternaria* can cause IL-25 release and tuft cell expansion in rhinosinusitis patients, but whether this pathway translates to healthy individuals or involves LTs is unclear [75].

## IV. Concluding remarks

**Fig 2:**
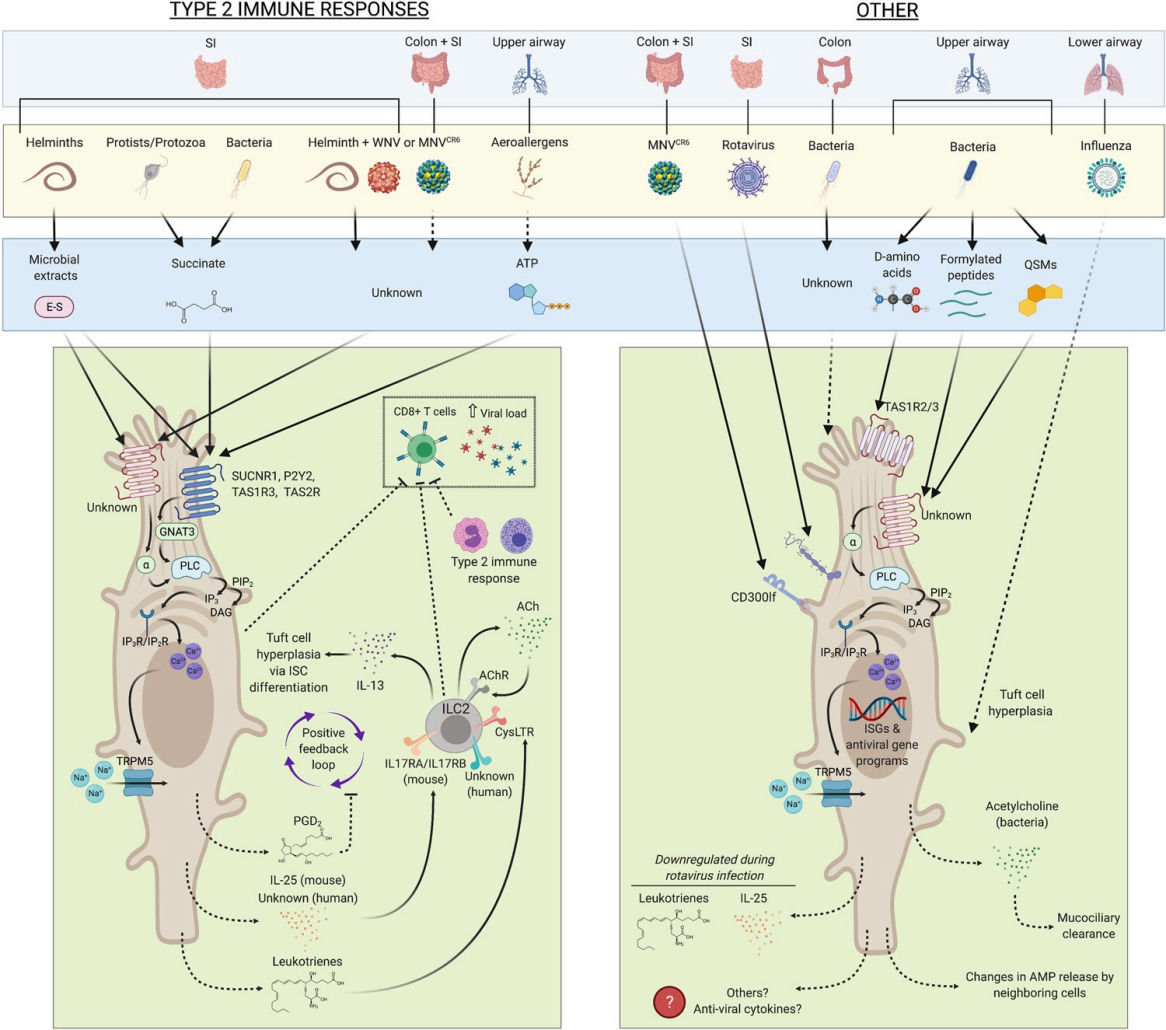
A current model of the sensor–effector pathways mediated by tuft cells in response to microbial stimuli. Dotted lines indicate intermediate or downstream pathways and indirect mechanisms (e.g., secretion mechanisms, CD8+ T cell ablation, or de novo tuft cell hyperplasia). ILC2 negative regulators (e.g., A20 and CISH) are not depicted for simplicity. Evidently, our understanding of tuft cell–microbial interactions and tuft cell responses are poorly characterized in the large intestine or outside of the context of type 2 immunity, especially in humans. ACh, acetylcholine; AMP, antimicrobial peptide; DAG, diacylglycerol; E–S, excretory–secretory; IL, interleukin; ILC2, type 2 innate lymphoid cell; MNV, murine norovirus; PGD, prostaglandin D; PLC, phospholipase C; QSM, quorum sensing molecule; TAS1R3, Taste 1 Receptor Member 3; TAS2R, Taste 2 Receptor; TRPM5, transient receptor potential cation channel subfamily M member 5; WNV, West Nile virus.

Tuft cells modulate a variety of host-pathogen interactions and act as key mediators of mucosal immunity to diverse microbes (Fig 2). Tuft cells can sense metabolic by-products, specific microbial components, and DAMPs using noncanonical pattern recognition receptors, including TAS1R3, SUCNR1 and P2Y2. In this sense, tuft cells function as immune sensory cells. Tuft cells can also maintain epithelial integrity by regulating commensal bacteria in the large intestine. With only a handful of ligands known to activate tuft cells and limited knowledge of downstream tuft cell effectors, there are likely others that have not been discovered. Overall, the role of tuft cells in orchestrating host microbial responses is highly context and location dependent, with unique activators and effectors depending on the luminal biome and tissue type. This likely has important implications between species, particularly in relating mouse tuft cell biology to that of humans. Given that luminal biomes, metabolites, and nutrient contents can vastly differ between mice and humans, we speculate that tuft cell function and behaviors in humans may be distinct. Perhaps human tuft cells sense different suites of luminal microbes or their by-products, resulting in unique downstream responses. To fully probe these differences, a scalable in vitro culture system for tuft cells will be needed to characterize the molecular mechanisms of tuft cell chemosensation and their downstream effector functions.

Tuft cells have known involvement in human cancers, but their specific roles and how tuft cells behave in other human diseases are relatively unexplored territories. With the recent discovery of thymic tuft cells in humans, the role of tuft cells in human tolerance and autoimmunity will likely bring critical insights into the human immune system [104]. As reduced helminth burdens and dysregulated microbiomes in human populations are correlated with increases in autoimmunity and allergy, it will be compelling to see whether tuft cells support anti-helminth immune responses or bacterial microflora in humans and how this relates to allergy and autoimmunity [123,124]. Finally, how tuft cells interact with human enteric viruses remains to be seen. Whether human tuft cells support viral infection in humans is unclear, but the prospect that tuft cells could offer an immune-privileged niche may have important consequences for chronic viral infections. Ultimately, tuft cells are a rare chemosensory cell type that facilitate striking interkingdom interactions between microbes and their hosts by integrating viral, bacterial, and parasitic pathogenesis and immunity.
